# MAGnitude-Image-to-Complex *K*-space (MAGIC-K) Net: A Data Augmentation Network for Image Reconstruction

**DOI:** 10.3390/diagnostics11101935

**Published:** 2021-10-19

**Authors:** Fanwen Wang, Hui Zhang, Fei Dai, Weibo Chen, Chengyan Wang, He Wang

**Affiliations:** 1 Institute of Science and Technology for Brain-Inspired Intelligence, Fudan University, Shanghai 200433, China; 19210850007@fudan.edu.cn (F.W.); hui_zhang@fudan.edu.cn (H.Z.); fdai@fudan.edu.cn (F.D.); 2 Philips Healthcare, Shanghai 200072, China; weibo.chen@philips.com; 3 Human Phenome Institute, Fudan University, Shanghai 201203, China

**Keywords:** deep learning, data augmentation, DICOM, raw data

## Abstract

Deep learning has demonstrated superior performance in image reconstruction compared to most conventional iterative algorithms. However, their effectiveness and generalization capability are highly dependent on the sample size and diversity of the training data. Deep learning-based reconstruction requires multi-coil raw *k*-space data, which are not collected by routine scans. On the other hand, large amounts of magnitude images are readily available in hospitals. Hence, we proposed the MAGnitude Images to Complex *K*-space (MAGIC-K) Net to generate multi-coil *k*-space data from existing magnitude images and a limited number of required raw *k*-space data to facilitate the reconstruction. Compared to some basic data augmentation methods applying global intensity and displacement transformations to the source images, the MAGIC-K Net can generate more realistic intensity variations and displacements from pairs of anatomical Digital Imaging and Communications in Medicine (DICOM) images. The reconstruction performance was validated in 30 healthy volunteers and 6 patients with different types of tumors. The experimental results demonstrated that the high-resolution Diffusion Weighted Image (DWI) reconstruction benefited from the proposed augmentation method. The MAGIC-K Net enabled the deep learning network to reconstruct images with superior performance in both healthy and tumor patients, qualitatively and quantitatively.

## 1. Introduction

Magnetic resonance imaging (MRI) is considered an important modality for clinical diagnosis, due to its strong capability in revealing the anatomy with different soft-tissue contrasts [1]. However, MRI is a slow acquisition process due to physical and physiological constraints. Raw MRI data are acquired sequentially to traverse the whole *k*-space. The prolonged scan time causes discomfort to the patients and results in severe motion artifacts in the reconstrued images.

A variety of accelerating techniques have been therefore proposed to shorten the scan time, among which Parallel Imaging (PI) [2,3] reduces the number of *k*-space samples acquired and recovers the images from aliasing artifacts. Historically, PI methods were put into two categories: approaches that operate in the image domain, inspired by SENSitivity Encoding (SENSE), and approaches that operate in *k*-space, inspired by GeneRalized Autocalibrating Partial Parallel Acquisition (GRAPPA) [4]. SENSE [2] uses spatial information from the coil sensitivity maps to solve the inverse problem recovering the images from aliasing artifacts. GRAPPA [3] uses linear shift-variant convolutional kernels to interpolate missing *k*-space lines using neighbouring acquired *k*-space lines. However, PI is limited to acceleration factors of two to three since a higher undersampling factor increases the noise level and residual aliasing artifacts [5]. Recently, deep learning has demonstrated great capability in accelerating MRI scans with higher undersampling factors. Zhu et al. [6] proposed an AUTOmated transform by Manifold APproximation (AUTOMAP) attempts to estimate the Fourier transform operation using fully connected layers, directly recovering the image without ever interpolating the missing *k*-space data. Wang et al. [7] trained a deep neural network to reconstruct aliasing-free images from undersampled ones in an end-to-end way. Hwan et al. [8] introduced a shift-invariant operator into the U-net [9], and obtained a mapping from under-sampled *k*-space to fully-sampled images. Lee et al. [10] applied prior knowledge from the phase-channel outcome into the reconstruction framework, achieving superior performance for magnitude-channel reconstruction. Yang et al. [11] incorporated *k*-space loss, content loss with adversarial loss, into a pix2pix [12] network to enforce similarity in both the frequency and image domains for reconstruction. Hammernik et al. [13] proposed a Variational Network (VN) to package image reconstruction into a regularization framework. Salman et al. [14] combined deep learned priors with SmooThness Regularization on Manifolds (SToRM) to capitalize local and population generalizable redundancies together with respiratory patterns. Aggarwal et al. [15] combined the power of data-driven learning with the Model-based framework using a Deep Learned prior (MoDL). Duan et al. [16] formulated the generalized PI reconstruction as an energy minimization problem and derived a variable splitting optimization method. Lv et al. [17] combined PI reconstruction with GAN to recover aliasing artifacts from undersampled *k*-space data, with an acceleration factor as high as six.

Although deep learning has proven potential in the field of medical image reconstruction beyond conventional methods, the effectiveness and generalization capability of deep learning-based reconstruction are highly dependent on the sample size and diversity of the training data. Severe overfitting problems may occur in cases of limited training samples or insufficient sampling [18]. Several data augmentation techniques have been proposed in the area of medical imaging. The most basic augmentation methods include shifting, rotation, shearing, and intensity perturbations [19]. Certain improvements were observed by introducing such variability in the training sets during lesion classification [20,21] and segmentation [19,22]. More advanced techniques have also been proposed. Using pix2pix translation GAN [19], Shin et al. [23] produced synthetic abnormal multi-parametric MRIs for tumor segmentation. Rusak et al. [24] used partial volume maps to guide a 3D GAN towards the generation of novel MRI volumes with more accurate tissue borders. Registration-based augmentation [25] benefits from subtle displacement and tissue characteristics captured in the existing dataset. Through modeling and applying deformation fields and additive intensity mask on existing labelled data, Chaitanya et al. [26] succeeded in generating more cases for the cardiac segmentation task. Abolvardi et al. [27] applied registration in a tumor case with a target from a healthy subject, thus generating new tumor cases with smoothly added lesions. Deriving displacement and appearance fields from pairs of brain images, Zhao et al. [22] developed a supervised deep learning model for image segmentation using one source case with a label.

Although the abovementioned augmentation algorithms are considered to have positive impacts on deep learning tasks thereafter, the reconstruction requires multi-coil raw *k*-space data. Existing studies mainly focused on magnitude image augmentations. However, the above data are not suitable for *k*-space augmentation, which is “parallelly” acquired with multiple coils and stored in a complex format. Collecting multi-coil complex data is not performed in clinical routine exams, which limits the reconstruction performance in most circumstances. On one hand, with the rapid development and popularization of MRI scanners, magnitude Digital Imaging and Communications in Medicine (DICOM) images are easily collected in hospitals.

Hence, we proposed the MAGnitude Images to Complex *K*-space (MAGIC-K) Net to generate multi-coil raw *k*-space data from existing magnitude images and a limited number of real *k*-space data to facilitate deep learning reconstructions. Taking reconstruction on DWI as an example, we succeeded in generating diversity with a larger *k*-space dataset. The MAGIC-K Net enabled the deep learning network for reconstruction to have superior performance in both healthy and tumor patients, qualitatively and quantitatively.

## 2. Materials and Methods

### 2.1. Data Acquisition

The current study was approved by the Institutional Ethics Review Board of our local institution. Informed consent was obtained from all subjects. A total of 30 healthy volunteers and 6 patients with different types of tumors, including lymphatic metastasis, diffused glioma, adenocarcinoma metastasis, temporal-lobe glioma, lymphatic metastasis, and benign acoustic nerve tumor, were recruited. All images as well as raw *k*-space data were acquired on a 3.0 T MRI scanner (Ingenia CX, Philips Healthcare; Best, the Netherlands), equipped with a 32-coil head coil. Multi-Shot DWI (MSDWI) was performed with a four-shot interleaved Echo Planar Imaging (EPI) sequence using the following parameters: Echo Time (TE), 75 ms; Repetition Time (TR), 2800 ms; matrix size, 228 × 228; FOV, 228 × 228 mm^2^; slice number, 16; slice thickness, 4 mm; slice gap, 1 mm; partial Fourier factor, 0.702; voxel size, 1.0 × 1.0 mm^2^. The MSDWI sequences consisted of *b*-values of 0 s/mm^2^ and 1000 s/mm^2^. The MSDWI data were reconstructed using MUSE [28] to eliminate phase aliasing. The Coil Sensitivity Maps (CSM) were estimated from the central *k*-space regions of each slice using ESPIRiT [29]. For the purpose of data augmentation, the T1-weighted (T1w) three-dimensional, Fourier-transformed acquisitions require Magnetization-Prepared 180 degrees radio-frequency pulses and RApid Gradient-Echo (MPRAGE) sequence was scanned with the following parameters: TE, 3.58 ms, TR, 8.05 ms; matrix size: 228 × 228; slice thickness, 1 mm; voxel size, 0.89 × 0.89 mm^2^.

Magnitude Digital Imaging and COmmunications in Medicine (DICOM) images from 26 healthy subjects were exported from the database of Fudan University. The MPRAGE images were obtained using identical scan parameters, as mentioned above.

### 2.2. MAGIC-K Net Architecture

**Figure 1:**
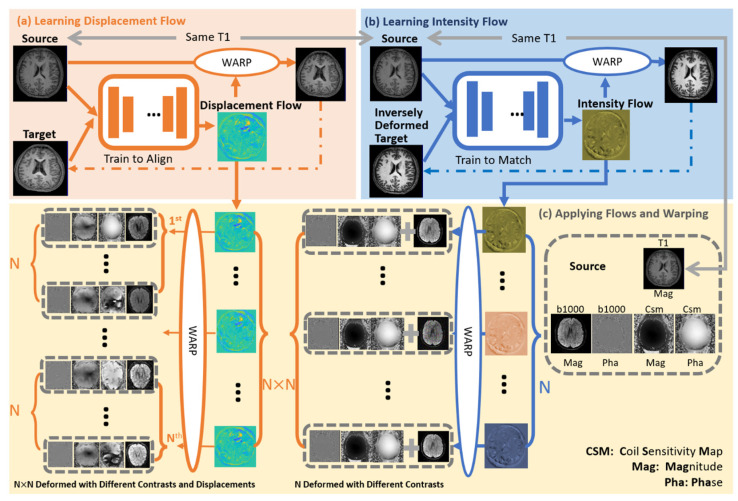
The architecture of the proposed MAGIC-K Net. The displacement and intensity flow fields were learned from pairs of T1w images. Then, the intensity and displacement flow fields were applied to the magnitude and phase of DWI with *b* = 1000 s/mm^2^, generating data sets with different contrasts and anatomical structures.

The architecture of the proposed MAGIC-K Net is shown in Figure 1, which contains three parts: (a) training of a geometrical model from source images to target ones, (b) training of an intensity model from source images to target ones, and (c) application of the pre-trained displacement and intensity flow fields to multi-coil complex images, for generating N × N raw images for reconstruction.

#### 2.2.1. MAGIC-K Net Training

Let $\left(S_{T},T_{T}\right)$ be two DICOM T1w data, defined over a 3D displacement domain $S_{T},T_{T}\subset {\mathbb{R}}^{3}$.

The displacement flow is in the form of a voxel-wise displacement field [30] of Φ=I+u, where u, the nonlinear misalignment between the volumes, can be learned, and $\Phi \subset {\mathbb{R}}^{4}$. To generate different brain structures, we warped $S_{T}$ to the *i*th target image $T_{T}^{i}$ using the following function:

(1) $$\Phi^{i}=C_{d}(S_{T},T_{T}^{i})$$

where $C_{d}$ represents the CNN to learn the displacement. After training the displacement network, it can be applied to the source images with the displacement flow field:

(2) $$\tau_{d}^{i}(S_{T})=S_{T}\circ \Phi^{i}$$

where $\tau_{d}^{i}$ stands for the displacement transformation from the source to the target.

Then, the inverse displacement flow of $C_{d}^{-1}$ was trained to transform the target to $S_{T}$, with the same anatomical structure but a different intensity. The pixel-wised intensity variation can be then trained using:

(3) $$\Psi^{i}=C_{i}(S_{T},C_{d}^{-1}(S_{T},T_{T}^{i}))$$

After training the intensity network, it can be applied to the source images as follows:

(4) $$\tau_{i}^{i}(S_{T})=S_{T}+\Psi^{i}$$

In this study, the architecture of the CNN was selected to be 3D-Unet [22]. The loss functions were defined as the Mean Squared Error (MSE) between the target and the outcome of the network:

(5) $$L_{d}(S_{T},T_{T}^{i})=\|T_{T}^{i}-S_{T}\circ \Phi^{i}{\|}^{2}$$

(6) $$L_{i}(S_{T},T_{T}^{i})=\|T_{T}^{i}\circ \Phi^{-i}-S_{T}+\Psi^{i}{\|}^{2}$$

where $\Phi^{-i}$ is the inverse displacement flow to transform the target to the source, $L_{d}$ stands for loss of the displacement network, and $L_{i}$ is the intensity loss.

#### 2.2.2. MAGIC-K Net Application

During the application of the MAGIC-K Net, DICOM T1w images were used for training. Since raw DWI images to be reconstructed are scanned in the same exam with T1w images, once the MAGIC-K Net has been trained, it can be directly applied to DWI images as well as CSMs. The intensity flow $\Psi^{i}$ and displacement flow $\Phi^{i}$ were applied to raw DWI source data $S_{D}$ and CSMs $S_{C}$, where $S_{D},S_{C}\subset {\mathbb{C}}^{3}$. The data in the complex format were separated into magnitude and phase components:

(7) $$S_{C}=S_{CM}\cdot e^{i\cdot S_{CP}}$$

(8) $$S_{D}=S_{DM}\cdot e^{i\cdot S_{DP}}$$

where $S_{CM},S_{CP},S_{DM},S_{DP}\subset {\mathbb{R}}^{3}$. Only the displacement flow was applied on $S_{CM},S_{CP},S_{DP}$ to keep the original intensities unchanged:

(9) $$\tau_{d}^{i}(S_{CP})=S_{CP}\circ \Phi^{i}$$

(10) $$\tau_{d}^{i}(S_{CM})=S_{CM}\circ \Phi^{i}$$

(11) $$\tau_{d}^{i}(S_{DP})=S_{DP}\circ \Phi^{i}$$

For the magnitude components of DWI, $\Psi^{i}$ and $\Phi^{i}$ were applied on $S_{DM}$ subsequently:

(12) $$\tau_{d}^{i}(\tau_{i}^{i}(S_{DM})=(S_{DM}+m\cdot \Psi^{j})\circ \Phi^{i}$$

where *i* and *j* stand for the *i*th and *j*th targets, m⊆ℝ stands for different scales of the intensity flows. In this study, m = 0.9, 1.1, 1.2, and 1.3, respectively, were used. Finally, the raw DWI sample size could be enlarged by N × N × m through this framework.

### 2.3. Training Data Augmentation

To better illustrate the value of the proposed MAGIC-K Net, only one case from a healthy volunteer was used as source images, including DICOM T1w images and the corresponding complex multi-coil DWIs. Another dataset containing DICOM T1w images from 26 subjects were used to train the MAGIC-K Net. For comparison, we applied basic and MAGIC-K Net augmentations as follows:

- shearing by 5%, 10%, −5%, and −10%;
- rotations of 20°, 40°, 60°, 80°, 100°, 120°, 140°, and 160°;
- translations along the *x* and *y* axes with 12, −12, 24, and −24 pixels;
- brightness with 5 scales (i.e., 0.8, 0.9, 1, 1.1, and 1.2 times of the average image intensity);
- MAGIC-K Net with displacement deformations (26 geometries) only;
- MAGIC-K Net with intensity variations (26 contrasts) and 4 scales (0.8, 0.9, 1.1, and 1.2).

Hence, we obtained 6 different augmentation types, as follows:

(s) BASIC: 4 (A) × 5 (D) = 20;
(r) BASIC: 8 (B) × 5 (D) = 40;
(t) BASIC: 16 (C) × 5 (D) = 80;
(s + r + t) BASIC: 4 (A) × 8 (B) × 16 (C) × 5 (D) = 2560;
(d) MAGIC-K: 26 (E) × 5 (D) ×16 (C) = 2080;
(d + i) MAGIC-K: 26 (E) × 26 × 4 (F) = 2730.

For each group, the training dataset contained 43,680 slices (16 slices for each case in the original dataset) after data augmentation for reconstruction. It is worth noting that no patients were included in the training dataset.

### 2.4. Deep Learning Reconstruction

MSDWIs from 30 healthy subjects and 6 patients with different tumor types were tested. The state-of-art VS-Net [16] was used for image reconstruction. Briefly, the VS-Net contained three blocks, including a denoiser block, a data consistency block, and a weighted average block, based on the multi-variable energy minimization process. Based on the notion of compressed sensing, VS-Net introduced auxiliary splitting variables to help enhance the fidelity of multi-coil data and simplify matrix inversion calculation.

We applied both uniform and variable density sampling trajectories with different undersampling rates (i.e., R = 4 and 6) for validation. The conventional iterative reconstruction SENSE [29] was also performed for comparison.

### 2.5. Performance Assessments

#### 2.5.1. Evaluation Metrics

The reconstruction performance was evaluated by the Peak Signal-to-Noise Ratio (PSNR) and Structure SIMilarity index (SSIM) [31].

#### 2.5.2. Apparent Diffusion Coefficient (ADC) Fitting

The ADC map [32] was obtained by fitting a pixel-wise mono-exponential function using DWI with different *b*-values:

(13) $$S_{b}/S_{0}=e^{-b\times ADC}$$

where $S_{b}$ is the magnitude signal of the high *b*-value DWI and $S_{0}$ is the magnitude signal with *b*-value = 0 s/mm^2^. The Brain Extraction Tool (BET) [33] was applied on DWI images and CSMs to strip the skull.

### 2.6. Model Implementation

We implemented the affine alignments between the paired data using SPM Co-registration and the DARTEL Toolbox [34].

Training and testing were implemented with Tensorflow [35] using 4 GPUs (4 cores P100, each with 16 GB memory). The network parameters were updated using the ADAM optimizer [36] with a fixed learning rate of 10^−3^ and a batch size of 40. The training iterations were set to 2000 for the (s) BASIC, (r) BASIC, and (t) BASIC groups, and to 10,000 for the (s + r + t) BASIC, (d) MAGIC-K, and (d + i) MAGIC-K groups. The training time was around 10 hours for the proposed (s + r + t) BASIC, (d) MAGIC-K and (d + i) MAGIC-K groups.

## 3. Results

**Figure 2:**
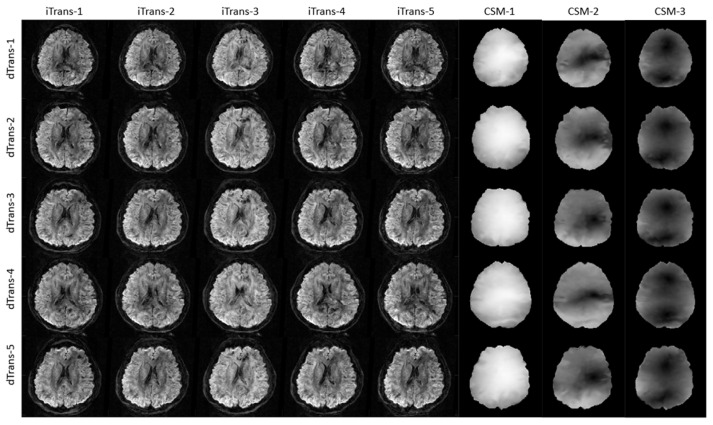
Cross-augmented high resolution DWIs with *b* = 1000 s/mm^2^ and the corresponding CSMs. The iTrans-N represents the intensity transformation from the Nth target. dTrans-N refers to the displacement from the Nth target.

Cross-augmented high resolution DWIs with *b* = 1000 s/mm^2^ and the corresponding CSMs are provided in Figure 2. The size of the training samples was increased, as well as the diversity of image distribution, which are crucial for the subsequent deep learning-based reconstruction network.

**Figure 3:**
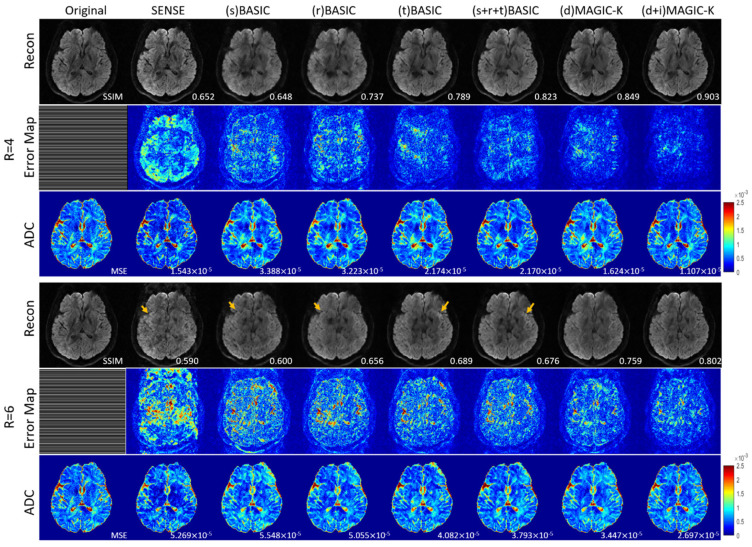
Comparison of the reconstructed images by different image augmentation strategies using uniform undersampling in a healthy volunteer. The SSIM is listed at the bottom-right of each reconstructed image. The error map was ×10 amplified for better visualization. The MSE is listed at the bottom-right of each ADC map. The aliasing artifacts are depicted by the yellow arrows.

**Figure 4:**
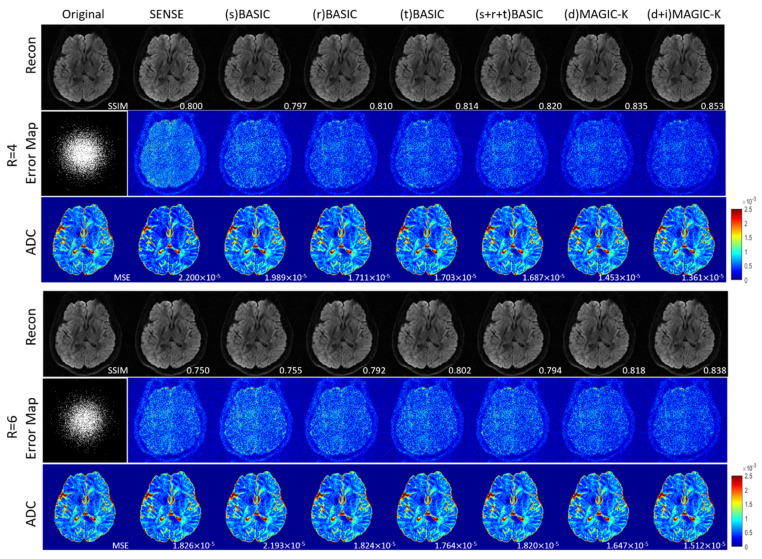
Comparison of the reconstructed images by different image augmentation strategies, using variable density undersampling in a healthy volunteer. The SSIM is listed at the bottom-right of each reconstructed image. The error map was ×10 amplified for better visualization. The MSE is listed at the bottom-right of each ADC map.

**Figure 5:**
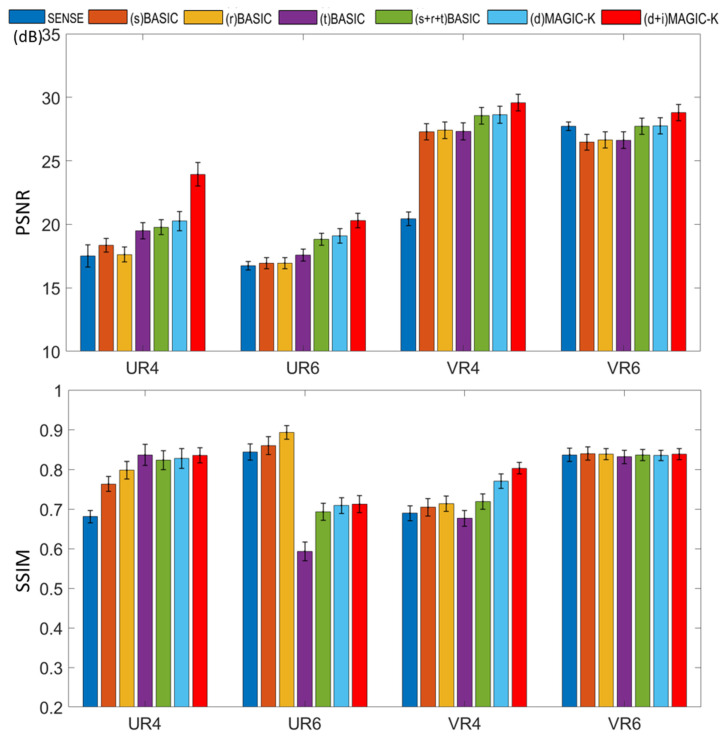
PSNR and SSIM of reconstructed images using different data augmentation strategies in healthy volunteers. U stands for the uniform undersampling strategy and V stands for the variable density undersampling strategy, and 4 and 6 stand for different acceleration factors, respectively.

**Figure 6:**
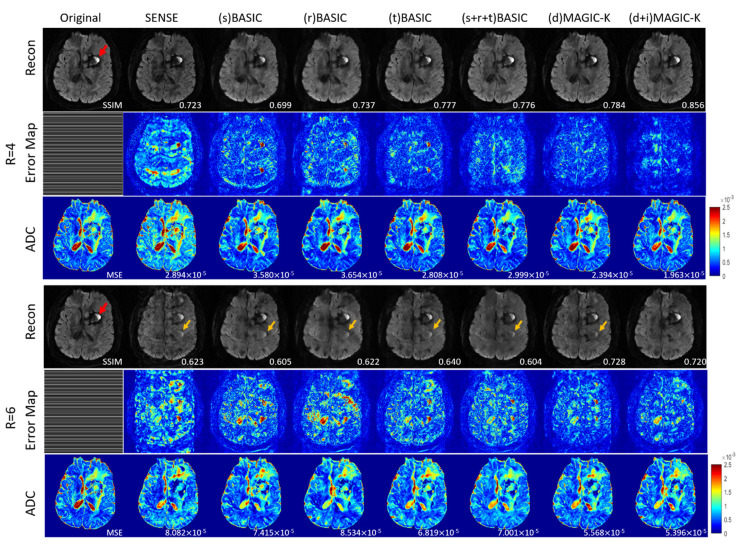
Comparison of the reconstructed images by different image augmentation strategies using uniform undersampling in a tumor patient with lymphatic metastasis. The SSIM is listed at the bottom-right of each reconstructed image. The error map was ×10 amplified for better visualization. The MSE is listed at the bottom-right of each ADC map. Aliasing artifacts are depicted by the yellow arrows. The tumor is depicted by a red arrow.

**Figure 7:**
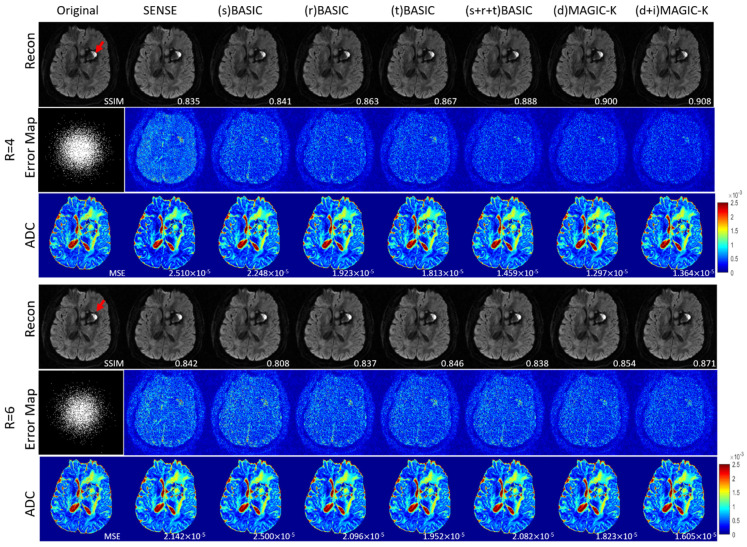
Comparison of the reconstructed images by different image augmentation strategies, using variable density undersampling in a tumor patient with lymphatic metastasis. The SSIM is listed at the bottom-right of each reconstructed image. The error map was ×10 amplified for better visualization. The MSE is listed at the bottom-right of each ADC map. The tumor is depicted by a red arrow.

**Figure 8:**
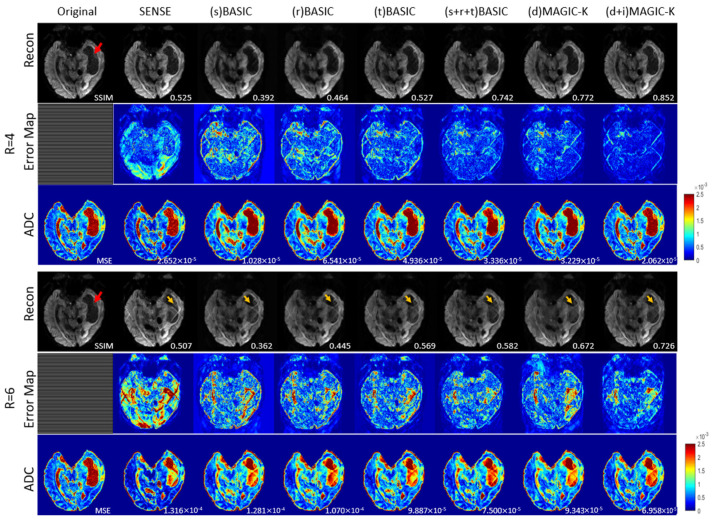
Comparison of the reconstructed images by different image augmentation strategies using uniform undersampling in a tumor patient with glioblastoma. The SSIM is listed at the bottom-right of each reconstructed image. The error map was ×10 amplified for better visualization. The MSE is listed at the bottom-right of each ADC map. Aliasing artifacts are depicted by the yellow arrows. The edema is depicted by a red arrow.

**Figure 9:**
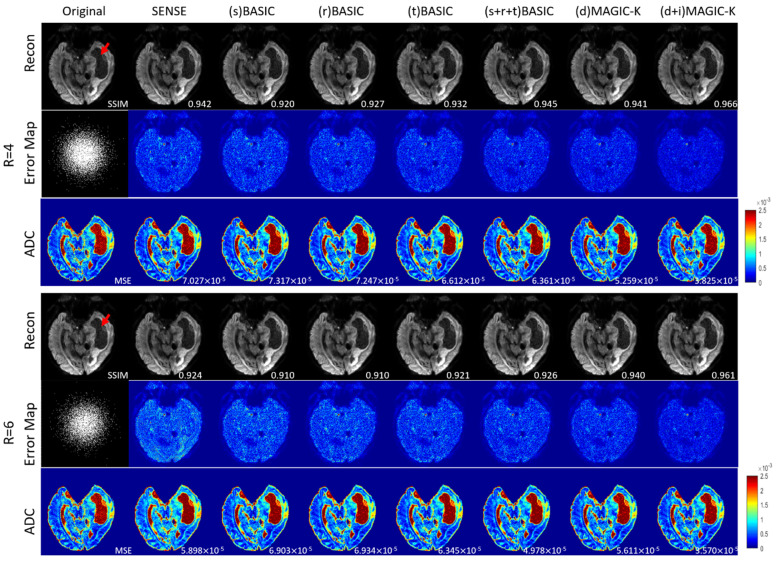
Comparison of the reconstructed images by different image augmentation strategies using variable density undersampling in a tumor patient with glioblastoma. The SSIM is listed at the bottom-right of each reconstructed image. The error map was ×10 amplified for better visualization. The MSE is listed at the bottom-right of each ADC map. Aliasing artifacts are depicted by the yellow arrows. The edema is depicted by a red arrow.

**Figure 10:**
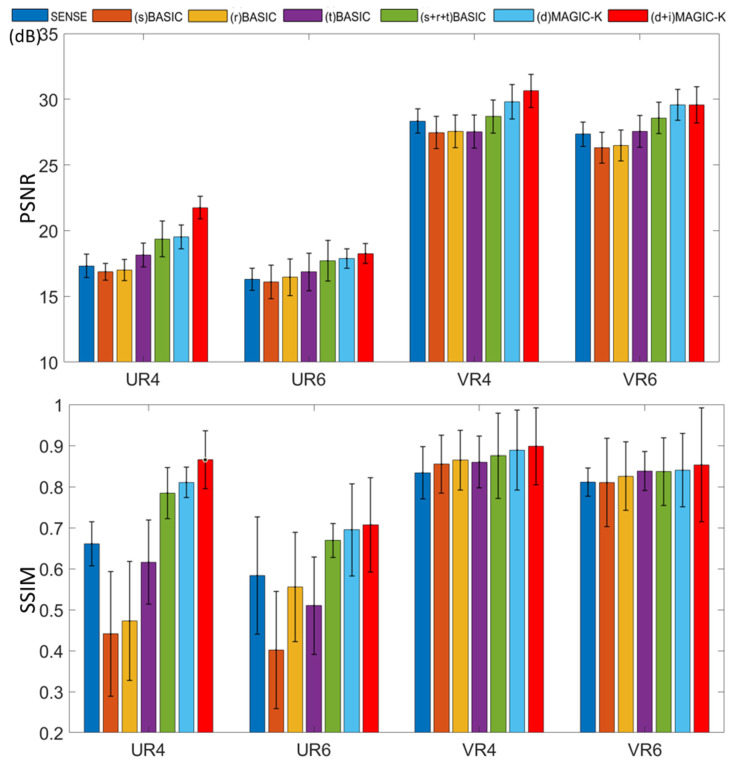
PSNR and SSIM of reconstructed images using different data augmentation strategies in tumor patients. U stands for the uniform undersampling strategy and V stands for the variable density undersampling strategy, and 4 and 6 stand for different acceleration factors, respectively.

The reconstruction results trained by different augmentation approaches are presented in Figure 3, Figure 4, Figure 5, Figure 6, Figure 7, Figure 8, Figure 9 and Figure 10. Tests on the healthy subjects are shown in Figure 3, Figure 4 and Figure 5 and tumor patients are shown in Figure 6, Figure 7, Figure 8, Figure 9 and Figure 10.

Compared with the (s) BASIC, (r) BASIC and (t) BASIC groups with data sizes of 20, 40, and 80, respectively, the (s + r + t) BASIC, (d) MAGIC-K and (d + i) MAGIC-K groups with data sizes of more than 2000 displayed higher PNSR and SSIM. (d) MAGIC-K, with a data size of 2080, displayed comparative outcomes to (s + r + t) BASIC, with a data size of 2560. Only local displacement transformations were applied to the original images. The (d + i) MAGIC-K Net achieved the highest PSNR (23.93 dB at R = 4, and 20.30 dB at R = 6) and SSIM (0.893 at R = 4, and 0.770 at R = 6). Moreover, aliasing artifacts remained using SENSE, as well as all the basic augmentations under uniform undersampling when R = 6, as depicted by the yellow arrows in Figure 3. However, both (d) MAGIC-K and (d + i) MAGIC-K succeeded in eliminating those aliasing artifacts.

When the undersampling trajectory was set to variable density, (d + i) MAGIC still displayed the highest SSIM (0.853) for reconstructed images and the smallest MSE (1.361 × 10^−5^) for ADC maps at R = 4, as well as the highest SSIM (0.838) for reconstructed images and smallest MSE (1.512 × 10^−5^) for ADC maps at R = 6 in Figure 4.

A representative patient with lymphatic metastasis is presented in Figure 6 and Figure 7. When the undersampling trajectory was set as uniform, (d + i) MAGIC-K provided the highest SSIM (0.876) at R = 4. Although displaying lower SSIM (0.720) at R = 6, (d + i) MAGIC-K performed better in eliminating aliasing artifacts in tumor regions compared with (d) MAGIC with a SSIM of 0.728, as depicted by the yellow arrows. Moreover, the MSE (5.396 × 10^−5^) between ADC maps calculated from the original and (d + i) MAGIC-K data was the smallest. For the variable undersampling trajectory that caused image blurring, (d + i) MAGIC-K displayed the highest SSIMs (0.908 at R = 4 and 0.781 at R = 6) for the reconstructed images.

Another representative tumor case with glioblastoma is presented in Figure 8 and Figure 9. Under the uniform undersampling trajectory, the (s) BASIC and (r) BASIC groups with small training data sizes failed to exceed the performance of SENSE (SSIM = 0.392 and 0.464 vs 0.525), demonstrating reduced generalization for tumor cases. At R = 6, the aliasing artifacts remained in all groups, as depicted by the yellow arrows. (d + i) MAGIC-K displayed the highest SSIM of 0.726 for the reconstructed images, with the corresponding lowest MSE (6.958 × 10^−5^) in ADC maps. The variable density undersampling induced blurring to low-resolution images, but (d + i) MAGIC achieved the highest SSIM of 0.961 at R = 6.

## 4. Discussion

In this study, we proposed a data augmentation solution to improve the image reconstruction task with limited amounts of training raw data, leveraging the diversity of DICOM images in hospitals. Different from other image processing tasks like segmentations or classifications, multi-coil raw *k*-space data were required to train a reconstruction network. However, it is often difficult to collect raw *k*-space data since they are not routinely obtained clinically. On the other hand, a large number of DICOM images are readily available in hospitals. The proposed MAGIC-K Net was able to generate large amounts of multi-coil raw *k*-space data based on DICOM images for reconstruction. Experimental results proved that the high resolution DWI reconstruction benefited from the proposed augmentation method; this can be explained by how the generalization performance of deep learning-based reconstruction relies heavily on the diversity of the training dataset. Although source images were from a healthy subject, the performance was quite stable in tumor patients.

Despite the availability of several public datasets of multi-coil raw data [37,38], the latter only focus on specific modalities (e.g., T1w, T2w, and T1w contrast-enhanced images). In contrast, the proposed MAGIC-K Net can enlarge datasets from any modality as long as anatomical DICOM images are acquired, which is applicable to most hospitals.

Compared with the basic data augmentation methods that apply global intensity and displacement transformations to the source images, the MAGIC-K Net can generate more realistic intensity variations and displacements from pairs of anatomical DICOM images. The training sample can be enlarged by N × N using N DICOM images. In addition, the MAGIC-K Net can be jointly used with basic global transformations to augment the training samples exponentially.

In this study, different sampling strategies have been studied. As the conventional sampling process in MRI, uniform undersampling poses a barrier to reconstruction since the associated low frequencies are not fully retained to preserve the overall structure; whereas, variable density undersampling creates incoherent aliasing artifacts, so that noise-like artifacts could be mitigated without degrading the structures. Compared the recoveries using the (d + i) MAGIC-K Net between UR6 and VR6, the tumor is still apparent in the reconstruction in UR6 (Figure 6) with SSIM = 0.720, while the structure reconstructed in VR6 (Figure 7) is well preserved with SSIM = 0.871. The SSIM of all testing patients using UR6 is 20.8% higher than the one of VR6 (0.854 vs. 0.707); hence, variable density sampling should be the preferable sampling strategy. However, for some special sequences, which are only practical using uniform undersampling, such as EPI, the proposed MAGIC-K Net is still sensible.

**Figure 11:**
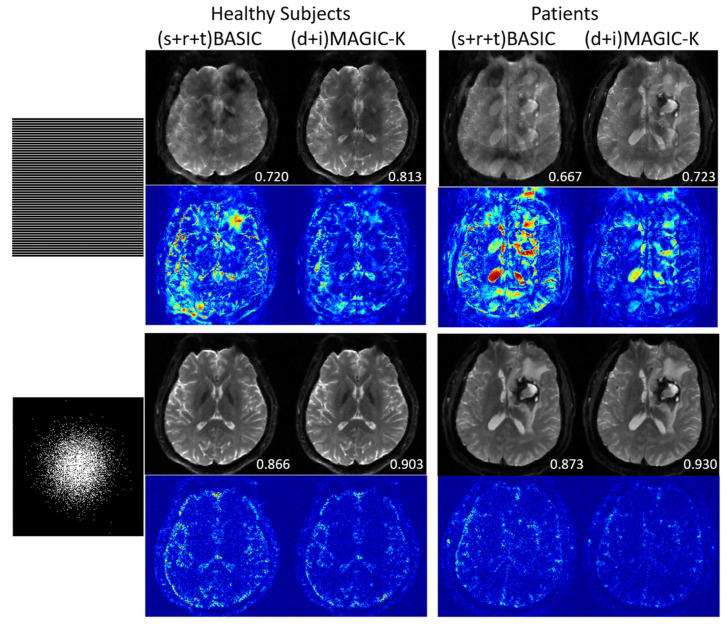
Comparison of the reconstructed images by data augmentation strategies of (s + r + t) BASIC and (d + i) MAGIC-K, using uniform undersampling with an acceleration factor of 6 (UR6) and variable density undersampling with an acceleration factor of 6 (VR6). The SSIM is listed at the bottom-right of each reconstructed image. The error map was ×10 amplified for better visualization.

We also evaluated the generalization of the proposed method on the dataset of fMRI. The clinical acquisition for fMRI mainly depends on the EPI collection, which is the same as DWI. Hence, we tested the proposed methods on the dataset of *b* = 0 s/mm^2^ DWIs for validation. The sampling strategy of uniform and variable density under a higher acceleration factor of 6 (UR6 & VR6) was tested. Theoretically, experiments on lower acceleration factors should have similar findings. The proposed method (d + i) MAGIC-K outperformed the basic method (s + r + t) BASIC in regard to PSNR and SSIM (Table 1 and Figure 11).

The limitations of this study should be mentioned. First, basic augmentation and MAGIC-K Net based only on displacement transformation displayed similar quantitative metrics (VR4, SSIM = 0.838 vs. 0.850 in healthy volunteers, with 0.889 vs. 0.899 in tumor patients). This emphasizes the undeniable role of basic methods in data augmentation. Since the augmentation only contains the displacement transformation, generalization of the network is limited. However, we observed a superior ability from the recovery of error maps (Figure 3). Second, for the variable density undersampling trajectory, which induces image blurring, the recovery ability of the network was quite similar. We may increase the acceleration factor [15] to differentiate the recovery ability of the networks. We tested the performance only under the acceleration factor of 4 and 6. To further evaluate the performance of the proposed data augmentation method, more tests under different acceleration factors [15] can be applied.

Several technical developments are still required in future studies. The number of source cases could be increased to further increase the training samples. Besides number increase, noise and motion could also be considered to improve the accuracy of the images. For a sequence with a long scan time, patient motion during the acquisition would affect the interpretability of images and degrade the performance of the algorithms. To better approximate non-ideal scenarios, both noise [39,40] and motion [41] could be incorporated into the images to better estimate the distribution of real MR data. Incorporating the uncertainty and generating heterogeneous data may add appearance variability and make deep learning methods more robust and generalizable to unknown data.

Augmentation of images from different patients is necessary to further improve the generalization performance. It is possible to validate the proposed method in other organs, e.g., abdomen [42], cardiac [43], knees [17], etc. It is also possible to apply the proposed augmentation method to unsupervised learning-based reconstructions when ground truth images are difficult to obtain.

## 5. Conclusions

This study proposed a new data augmentation method to generate multi-coil *k*-space data for deep learning-based image reconstruction. Leveraging high magnitude DICOM images in hospitals, we succeeded in increasing the size and diversity of the training dataset. The reconstruction trained with the data generated by the proposed MAGIC-K Net outperformed routine data augmentation methods and demonstrated better generalization to patients with different types of brain tumors. The MAGIC-K Net can be further applied to accelerate the detection and diagnosis of brain tumor patients.

## Figures and Tables

**Table 1:** PSNR and SSIM of the reconstructed images using data augmentation strategies of (s + r + t) BASIC and (d + i) MAGIC-K. The tests were applied on the healthy subjects and tumor patients. R stands for the acceleration factor.

**Uniform Undersampling with R = 6.**		**PSNR (dB)**	**SSIM**
Healthy Subjects	(s + r + t) BASIC	21.093 ± 0.789	0.694 ± 0.044
	(d + i) MAGIC-K	23.901 ± 0.632	0.764 ± 0.043
Patients	(s + r + t) BASIC	19.234 ± 0.734	0.683 ± 0.031
	(d + i) MAGIC-K	21.417 ± 0.693	0.715 ± 0.043
**Variable Density Undersampling with R = 6**		**PSNR (dB)**	**SSIM**
Healthy Subjects	(s + r + t) BASIC	30.432 ± 0.453	0.859 ± 0.033
	(d + i) MAGIC-K	32.954 ± 0.581	0.903 ± 0.028
Patients	(s + r + t) BASIC	29.043 ± 0.734	0.844 ± 0.031
	(d + i) MAGIC-K	31.890 ± 0.843	0.913 ± 0.024

## Data Availability

The data presented in this study are available upon request from the corresponding author. The data are not publicly available due to the patients’ right to privacy.
